# A 56 year old woman with clinically significant p.Arg121Gln-/R92Q TNFRSF1A mutation

**DOI:** 10.1186/1546-0096-13-S1-P44

**Published:** 2015-09-28

**Authors:** I von Mühlenen, B Claviezel

**Affiliations:** 1 Rheuma-Basel, Rheumatology Office, Basel, Switzerland; 2 Dornach Spital, Internal Medicine, Dornach, Switzerland

## Background

Tumor necrosis factor receptor-associated periodic syndrome (TRAPS) is a rare autoinflammatory disorder caused by TNFRSF1A mutations. The symptoms of TRAPS are various and often unspecific. The R92Q variant is considered to be a low penetration mutation with mild phenotype.

## Objective

To analyze the clinical course of a woman carrying the R92Q Mutation and presenting with rheumatic disease and some symptoms consistent with autoinflammatory syndrome.

## Methods

A woman was identified as carrying the p.Arg121Gln-/R92Q mutation in the TNFRSF1A gene and presenting with autoinflammatory symptoms.

## Results

The patient was hospitalized 3 times with abdominal pain elevated CRP and fever. She was presumed to have recurrent abdominal infections and treated with antibiotics. The laboratory tests showed no increase on leukocytes counts but elevated CRP (to198mg/l), which normalized after a few days. CT-scans and 2 ultrasounds were performed, which showed signs of panniculitis of the abdominal fat, but no other pathology. Even a diagnostic laparoscopy was performed with no further results. The patient had a history of recurrent fever (39°C), arthralgia, myalgia, abdominal pain, diarrhoe, backpain, hyperkeratotic skin lesions and constantly elevated ESR and CRP. Interestingly, ANA and dsDNS antibodies had been showed to be elevated over many years. The patient was first diagnosed an overlap connective tissue disease/undifferentiated SpA. Later on, periodic fever syndrome was suspected and the genetic tests performed. The genetic analysis showed the presence of p.Arg121Gln-/R92Q mutation. When treated with methorexate and etanercept the patient showed improvement of most symptoms including arthralgia, back pain and skin disease. ANA and dsDNS antibodies turned negative but CRP and ESR remained elevated. The fever episodes were persisting so that etanercept was stopped and the treatment with IL-1 antagonist startet. Under the treatment with anakinra 100 mg every second day, we observed an improvement of fever and abdominal symptoms although no remission. The skin lesions, the arthralgia and back pain returned. Anakinra dosis was enhanced to daily applications of 100 mg with further improvement of the autoinflammatory symptoms. Back pain, arthralgia and skin lesions persist.

## Conclusion

Our findings suggest that the R92Q mutation is influencing the phenotype of the rheumatic disease in this patient and seams to be responsible for part of the presenting symptoms.

## Consent to publish

Written informated consent for publication of their clinical details was obtained from the patient/parent/guardian/relative of the patient.
